# Measuring cost and affordability of current vs. healthy diets in Argentina: an application of linear programming and the INFORMAS protocol

**DOI:** 10.1186/s12889-021-10914-6

**Published:** 2021-05-10

**Authors:** Gabriel Giacobone, Maria Victoria Tiscornia, Leila Guarnieri, Luciana Castronuovo, Sally Mackay, Lorena Allemandi

**Affiliations:** 1 FIC Argentina, Arévalo 2364 1A, 1425 CABA, Argentina; 2 School of Population Health, University of Auckland, Private Bag 92019, Auckland, 1142 New Zealand

**Keywords:** Costs and cost analysis, Noncommunicable diseases, Obesity, Public health, Fiscal policy, Nutrition policy, Diet

## Abstract

**Background:**

Food cost and affordability is one of the main barriers to improve the nutritional quality of diets of the population. However, in Argentina, where over 60% of adults and 40% of children and adolescents are overweight or obese, little is known about the difference in cost and affordability of healthier diets compared to ordinary, less healthy ones.

**Methods:**

We implemented the “optimal approach” proposed by the International Network for Food and Obesity/non-communicable diseases Research, Monitoring and Action Support (INFORMAS). We modelled the current diet and two types of healthy diets, one equal in energy with the current diet and one 6.3% lower in energy by linear programming. Cost estimations were performed by collecting food product prices and running a Monte Carlo simulation (10,000 iterations) to obtain a range of costs for each model diet. Affordability was measured as the percentage contribution of diet cost vs. average household income in average, poor and extremely poor households and by income deciles.

**Results:**

On average, households must spend 32% more money on food to ensure equal energy intake from a healthy diet than from a current model diet. When the energy intake target was reduced by 6.3%, the difference in cost was 22%. There are no reasonably likely situations in which any of these healthy diets could cost less or the same than the current unhealthier one. Over 50% of households would be unable to afford the modelled healthy diets, while 40% could not afford the current diet.

**Conclusions:**

Differential cost and affordability of healthy vs. unhealthy diets are germane to the design of effective public policies to reduce obesity and NCDs in Argentina. It is necessary to implement urgent measures to transform the obesogenic environment, making healthier products more affordable, available and desirable, and discouraging consumption of nutrient-poor, energy-rich foods.

## Background

The most recent National Risk Factor Survey in Argentina [1] indicates that over 60% of the adult population suffers from excess weight, while only 6% of the population meet fruit and vegetable intake requirements. Additionally, over 40% of children and adolescents between 5 and 17 years old are overweight or obese [2]. In this context, promoting healthy eating habits and the consumption of less energy-dense foods is critical to reduce the prevalence of non-communicable diseases (NCDs) associated with excess weight.

One of the main causes of the obesity epidemic is the obesogenic environment, defined as ‘the sum of influences that the surroundings, opportunities, or conditions of life have on promoting obesity in individuals or populations’ [3], including those that facilitate excessive energy intake. While there are many factors affecting eating habits, the cost and affordability of healthy diets -i.e. the proportion of household income needed to buy the food products- has been underscored as one of strongest barriers against healthier eating at the population level, perhaps exerting an even more powerful influence on food choice than taste, promotions, and convenience [4–7].

Many studies have attempted to broach the issue of healthy vs. unhealthy eating costs, with varying methodologies and results. Some studies have compared pricing data of healthy/less healthy versions of specific product categories (e.g. processed vs. unprocessed meat), while others have focused on the total price of complete diets for reference households or diet patterns [8]. Conflicting results may also arise from the different units used to measure the cost of food. For example, Carlson and Frazão [9] found that healthier foods cost less than less healthy foods in the USA when measured in price by edible weight ($/100 g) or average portion ($/average portion), but not when measured as price by energy unit ($/kcal). This could be explained by the fact that healthier foods tend to be less energy-dense (e.g. fruit, vegetables) and will thus be more expensive per energy unit than highly energy-dense foods [10].

A typical Argentinean diet is not diverse, characterised by high consumption of red meat and very low consumption of fruit and vegetables. Although this is true for the entire population [11], there is strong evidence that people with lower socioeconomic status in Argentina consume significantly less fruit, vegetables and dairy and higher amounts of soft drinks and confectionery as compared to the rest of the population [12]. Despite the unquestionable importance of this information to advocate for effective public health policies targeting environmental determinants of excess weight, evidence on the differential price and affordability of healthier diets is very limited in Latin America with a few notable exceptions [13–15]. This study aimed to bridge this information gap in Argentina by estimating the cost differential and affordability of the current diet vs. healthy diets as of 2018, using the data collection and analysis approach proposed by the International Network for Food and Obesity/non-communicable diseases Research, Monitoring and Action Support (INFORMAS) [16].

## Methods

### The INFORMAS optimal approach

INFORMAS is a global network of organizations and researchers committed to the promotion of healthy food environments worldwide, with the ultimate goals of reducing obesity, diet-related NCDs and their related inequalities. The INFORMAS protocol [17] provides guidelines to systematically collect and analyse information on diet cost and affordability in a reproducible and comparable way. It describes three different approaches: a) the food approach (or “minimal” approach) to monitor the cost of healthier and less healthy food products over time, b) the meals approach (“expanded approach”) that compares and monitors the cost of popular takeaway meals compared to healthier home-cooked counterparts; and c) the diet approach (“optimal approach”), which involves the design of model current and healthy diets for reference households for a defined period of time, in view of specific nutritional targets, drawing from local dietary guidelines and data on the most popular food products consumed by the population, as well as obtaining household income data to evaluate affordability of healthy diets. This work is based on the optimal approach of INFORMAS, enhanced by linear programming to model current and healthy diets, as detailed below.

### Definition of “current” and “healthy” diets

Model diets were developed considering a typical household structure, a 45-year-old man and woman, 14-year-old boy and 7-year-old girl, for a period of 2 weeks as recommended by the INFORMAS framework [16].

The model current diet (CD) was based on the most commonly consumed foods, defined as those products that were purchased by at least 5% of households as reported by the National Household Expenditure Survey 2012–2013 (ENGHo) [18], an approach that has been used in another study also based on the INFORMAS protocol [19]. As the protocol suggests, common foods should be culturally acceptable, commonly eaten and widely available.

Once the most commonly consumed foods were identified, they were grouped as per the Dietary Guidelines for the Argentinean Population (GAPA) [20] into: fruit and vegetables; legumes, cereals, potatoes, bread and pasta; milk, yoghurt and cheese; meat and eggs; oils, nuts and seeds; and discretionary foods. These groups were further subdivided into categories to achieve higher specificity of our diet cost model. Despite not being included in GAPA food groups, non-sugary beverages, alcohol beverages and salt were also considered to model CDs because they were part of the most consumed foods [21]. The ENGHo also provides an estimate of the amount of specific foods (g) consumed by an “equivalent adult”, a unit that standardizes energy requirement to that of an adult male 30 to 60 years-old (2.750 kcal/day). The amount of different food groups (g) consumed by a typical household were estimated in reference to this standard unit, following the equivalent adult conversion table provided by the National Statistics and Census Institute (INDEC) for males and females of different ages [21] and resting on the assumption that all household members consume the same foods but in different quantities. Total energy (kcal), carbohydrates (g), total sugars (g), sugars (g), total fat (g), saturated fat (g), protein (g), fiber (g) and sodium (mg) intake were calculated based on these food amounts, using nutrient content data from a previous study [22].

The healthy diet (HD) was also designed on the basis of the most consumed foods but including a greater variety of foods with better nutritional quality, such as lean meats and fish, wholemeal bread and cereals, and low-fat dairy products. These selections were performed in view of the nutrient and food group targets set forth by the UN’s Food and Agriculture Organization and World Health Organization (FAO/WHO) nutritional standards [23–25] and GAPA [20].

Since it is very difficult to design model diets meeting these targets exactly, minimum and maximum constraints for each specified food group and nutrient content were established as per the INFORMAS DIETCOST programme rationale [26], that allows for a +/− 30% margin for nutrient requirements and +/− 1.5% for energy yields. Energy intake targets were kept constants for both the CD and HD model diets.

Nutrition professionals in our research team elaborated a two-week menu (including breakfast, lunch, afternoon snack and dinner) for the typical household and calculated the amount for each food category necessary to achieve energy (kcal), macronutrients (g), fiber (g) and sodium (g) intake levels consistent with ENGHo estimates for current intake by food group (CD) or meeting nutritional targets (HD). This process resulted in a list of weight and energy yields for 112 different products belonging to 28 food categories, included in different proportions in CD and HD (Table 1).

**Table 1:** Weight and energy yield targets for two-week model current diet (CD), equal-energy (HD) and reduced-energy healthy diet (HD2)

Food category	CD		HD		HD2	
	Weight (g)	Energy (kcal)	Weight (g)	Energy (kcal)	Weight (g)	Energy (kcal)
Fruit	6300.0	3219.0	19,180.0	9193.0	17,746.5	8505.9
Non-starchy vegetables	7350.0	2543.0	22,820.0	7152.0	21,114.5	6617.0
White bread	7700.0	19,834.0	1540.0	3967.0	1424.9	3670.3
Starchy vegetables	6916.0	5785.0	700.0	5855.0	6476.8	5417.4
Cereal	7840.0	23,801.0	2310.0	7013.0	2137.4	6489.4
Legumes	0.0	0.0	4970.0	13,135.0	4598.6	12,153.6
Wholemeal bread	0.0	0.0	4620.0	11,59	4274.7	10,722.4
Wholemeal cereal	0.0	0.0	4690.0	16,79	4339.5	15,534.6
Milk	5152.0	2607.0	19,81	8881.0	18,329.4	8217.2
Low-fat yogurt	0.0	0.0	2450.0	305.0	2266.9	964.9
Regular yogurt	1680.0	1138.0	910.0	616.0	842.0	570.3
Cheese	1820.0	6381.0	420.0	1472.0	388.6	1362.1
Low-fat cheese	0.0	0.0	560.0	1101.0	518.2	892.8
Eggs	2800.0	4362.0	2240.0	3,49	2072.6	3229.1
Meat	6300.0	10,641.0	6230.0	8456.0	5764.4	7827.9
Vegetable oil	1050.0	9,45	1400.0	12,6	1295.4	11,658.3
Fatty foods	560.0	2505.0	490.0	2192.0	453.4	2027.6
Cookies	1540.0	6637.0	560.0	2413.0	518.2	2233.1
Processed meat	1600.0	3548.0	280.0	621.0	259.1	573.9
Sweets	1890.0	4396.0	490.0	1608.0	453.4	1488.1
Bouillon cubes and powders	3500.0	147.0	168.0	12.0	155.4	11.1
Snacks	980.0	4903.0	196.0	905.0	181.4	837.4
Sugary beverages	11,200.0	5280.0	280.0	76.0	259.1	70.3
Sugar	1050.0	4200.0	280.0	1120.0	259.1	1036.3
Salt	196.0	0.0	0.0	0.0	0.0	0.0
Non-sugary beverages	6892.0	20.0	6892.0	20.0	6376.9	18.5
Alcohol beverages	1260.0	832.0	0.0	0.0	0.0	0.0

Finally, a second healthier diet -HD2- was modelled by reducing the total energy yield of the HD diet. Although the original aim was to an 8% energy reduction, as has been done in comparable studies [19, 27], it was not possible to do so and still meet food category weight and energy yield targets. Thus, the HD2 -reduced-energy healthy diet- represented a 6.3% energy reduction vs. CD/HD. These reductions were conducted across all food groups to maintain the same relative energy participation per food group as the HD. For example, if fruit represented 10% of the total energy yield in HD, then it also accounted for 10% of the HD2 (Table 1).

The model diets were designed using a linear programming (LP) routine using Microsoft Excel’s Solver. Instead of using LP as an optimization tool (i.e. to minimize/maximize an objective function), we applied this method to obtain diets that were combinations of the 112 food products (*g*_*ij*_) and met the weight targets (${\overline{g}}_j$) for each of the 28 food categories (*j*) as per eq. (1)while simultaneously meeting energy yield constraints for each category, as per eq. (2). Weight and energy targets for each food category are shown in Table 1. Since the macro- and micro-nutrient targets are already considered in the design of food group intake targets as per GAPA and WHO/FAO recommendations, our model did not explicitly include nutrient content thresholds.

1 $$ \sum \limits_{i,j=1}^n{g}_{ij}={\overline{g}}_j\forall j $$

2 $$ \sum \limits_{i,j=1}^n{Kcal}_{ij}={\overline{Kcal}}_j\forall j $$

We ran the model three times for each diet type to obtain three different product combinations for each. The resulting nine model diets (three per diet type: CD, HD, and HD2) were controlled and modified if necessary by our nutrition professionals to ensure that the quantities obtained for each product made sense from a nutritional and cultural point of view and to have an adequate representation of each food category according to their frequency of consumption.

### Product price and diet costs

In order to ensure maximum reliability of the information, prices for all the products included in both diets as of September 2018 were estimated from official and non-official data sources, including the National Institute of Statistics and Census’ (INDEC) ENGHo (adjusted for inflation, for 21% of the products) and Consumer Price Index (CPI, 38%), as well as the City of Buenos Aires’ CPI (17%) and private consultants (24%).

There is currently no consensus in the literature as to what price metric is best to use [8, 28, 29], so two price metrics were considered: price per weight ($/gram); and price per energy unit ($/kcal) to obtain complementary results.

Diet costs were estimated through Monte Carlo simulations performed for each of the diet types designed in the previous step, assuming that diet cost was normally distributed with mean and standard deviation equal to the cost average and standard deviation of the three diet options obtained by the linear model. The simulations included 10,000 iterations per diet type (CD, HD and HD2), resulting in 30,000 combinations of product options and concurrent diet costs for a typical household over a two-week period.

### Affordability

Affordability analysis consists of comparing purchase costs to available monetary resources. Using data from INDEC’s Permanent Household Survey (EPH) [30] we calculated the participation (%) of the CD, HD and HD2 cost in the average reference household income for all households, for poor and extremely poor households, and per household income deciles.

EPH data from the second semester of 2018 indicate that, on average, households spend 40% of their income on food [30]. Allowing for a 25% sensitivity margin, diets could be considered affordable if they represented 50% or less of the total household budget.

## Results

### Cost of diets

For the first approach, when HDs and CDs presented the same energy value (119,797.6 kcal ±1.5%) the estimated average cost of HDs was 31.7% higher than the CDs (AR$ 7453 vs. AR$ 5659). When normalized to product weight ($/100 g), no significant difference was found between diet costs (Table 2).

**Table 2:** Cost of the two-week model current diet (CD), equal-energy (HD) and reduced-energy healthy diet (HD2) by Monte Carlo simulation (10,000 iterations)

Model diet (10,000 iterations)	Cost (AR$)					Mean per 100 net g
	Mean	Median	SD	Range	Mean per 100 Kcal	
**CD**	5658.7	5659.0	87.0	5388.0–5920.0	4.7	6.7
**HD**	7453.2	7454.0	123.0	7012.8–7773.0	6.1	6.7
*Diff.* vs. *CD*	*+ 31.7%*					
**HD2**	6892.8	6873.1	199.0	6134.0–7655.0	6.2	6.7
*Diff.* vs. *CD*	*+ 21.8%*					

In the second approach (HD2), where the model healthy diets had 6.3% less energy than the CDs (119,797.6 kcal vs 112, 268.0 kcal), the average total cost was estimated at AR$ 6894, a 21.8% higher than the average CD cost. No significant difference was observed when cost was normalized to product weight (Table 2).

**Fig. 1:**
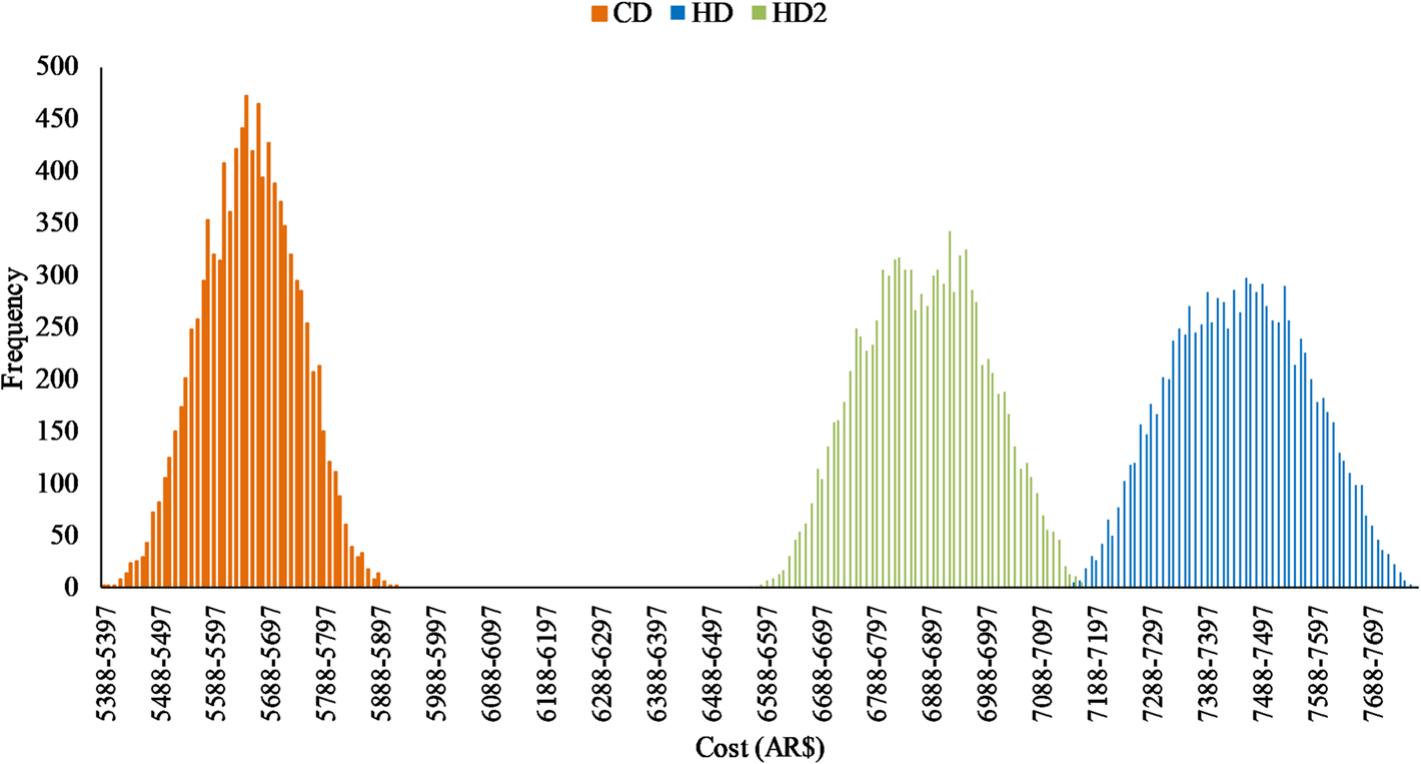
Distributions for the two-week cost (AR$) of model current diets (CD), equal-energy (HD) and reduced-energy healthy diets (HD2) by Monte Carlo simulation (10,000 iterations)

As shown by Fig. 1, there were no superpositions in the diet cost distributions for CDs, HDs and HD2s.

The proportion of energy provided by each food is similar for both healthy diets (HD and HD2), which explains the same cost proportion by food group. Products with lower cost per energy unit represented a higher proportion of the total cost of the CDs compared to HDs and HD2s, such as white bread (8.2% vs. 1.2%), sugary beverages (6,8% vs 0.1%), and other discretionary foods: fatty foods, cookies, processed meat, sweets, snacks and sugar (11.1% vs 1.8%). Inversely, more expensive products per energy unit represented a much higher proportion of the total product basket cost for the HDs than the CDs, such as fruit and non-starchy vegetables (36.5% vs. 13.2%) (Table 3).

**Table 3:** Energy and cost proportion per food category of model current diets (CD), equal-energy (HD) and reduced-energy healthy diets (HD2)

Food category	% of total energy		% of total cost	
	CD	HD/ HD2	CD	HD/ HD2
Fruit	2.7	7.6	5.3	12.3
Non-starchy vegetables	2.1	5.9	7.9	24.1
White bread	16.5	3.3	8.2	1.2
Starchy vegetables	4.8	4.8	9.5	5.2
Cereal	19.8	5.8	4.4	1.0
Legumes	0.0	10.8	0.0	5.0
Wholemeal bread	0.0	9.6	0.0	3.9
Wholemeal cereal	0.0	13.8	0.0	2.3
Milk	2.2	7.4	2.9	8.0
Low-fat yogurt	0.0	0.9	0.0	1.0
Regular yogurt	0.9	0.5	0.8	0.3
Cheese	5.3	1.2	3.5	0.5
Low-fat cheese	0.0	0.8	0.0	1.4
Eggs	3.6	2.9	3.5	2.1
Meat	8.9	7.0	29.0	24.3
Vegetable oil	7.9	10.4	1.4	1.7
Fatty foods	2.1	1.8	0.5	0.3
Cookies	5.5	2.0	0.7	0.2
Processed meat	3.0	0.5	7.6	0.8
Sweets	3.7	1.3	1.1	0.3
Bouillon cubes and powders	0.1	0.0	0.0	0.0
Snacks	4.2	0.9	0.7	0.1
Sugary beverages	2.5	0.1	6.8	0.1
Sugar	3.3	0.9	0.5	0.1
Salt	0.0	0.0	0.1	0.0
Non-sugary beverages	0.0	0.0	4.5	3.4
Alcohol beverages	0.7	0.0	1.2	0.0

### Affordability

**Fig. 2:**
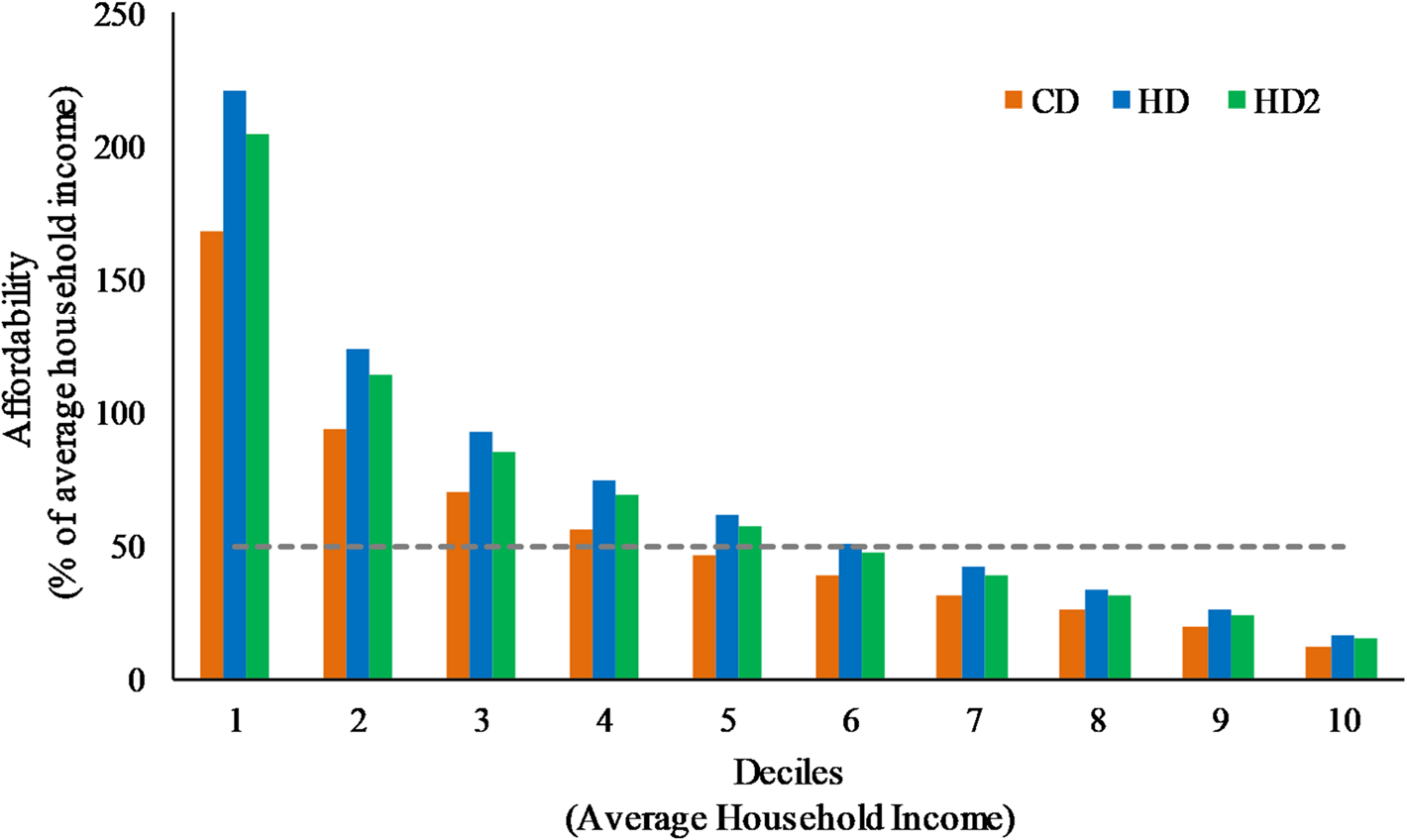
Affordability of model current diets (CD), equal-energy (HD) and reduced-energy healthy diets (HD2) by household income deciles. The dashed line indicates the affordability threshold (50%)

CD cost represented 33.4% of the average household income overall, compared to 44.1% for the first approach, equal-energy HD and 40.7% for the second, reduced-energy HD2. For poor households, proportions were much higher, ranging from 76.1% for CD to 100.2% of average household income for HD and 92.5% for HD2. All modelled diet costs were far above the average household income of extremely poor households (Table 4). Figure 2 depicts affordability of the three types of modelled diets according to income deciles. Considering 50% of average household income as a threshold for affordability, on average CDs were considered affordable by 60% of the total households, while only 40% of households could afford HDs and the 50% could afford HD2s.

**Table 4:** Affordability of model current diets (CD), equal-energy (HD) and reduced-energy healthy diets (HD2) in average, poor and extremely poor households

Households	Average Income (in AR$)	Affordability (% of income)		
		CD	HD	HD2
**Average**	33,839	33.4	44.1	40.7
**Poor**	14,872	76.1	100.2	92.5
**Extremely poor**	6116	185.0	243.7	224.9

## Discussion

This is the first study to compare the cost of healthy vs. current diets in Argentina using the optimal approach recommended by INFORMAS. The results suggest that, on average, a reference household must spend 31.7% more money on food to achieve the same total energy intake from a healthy diet than from a diet modelled on current eating patterns. The lack of overlap in diet price distributions suggest that there are no reasonably likely situations in which healthier diets could cost less or the same as the current unhealthier one.

This result is consistent with other studies following the INFORMAS protocol that have found healthy diets to be more expensive by the calorie, such as New Zealand (8% difference in cost) [19] and Sweden [31]. Moreover, our results also support the claim that energy-dense diets are less expensive than healthier diets, as has been shown in France [32]. Additionally, a meta-analysis of studies from around the world has shown that healthy diets are $1.15 to $1.94 more expensive per day than less healthy diets when standardized to a daily 2000 kcal intake. When measured in price by edible weight (AR$/100 g), the differences among modelled diets were negligible.

When the total energy in the healthy diet was reduced by 6.3%, the cost gap between the healthy diet and current diet was also reduced, from 31.7 to 21.8%. This finding is relevant in the context of facilitating weight loss, since one of the main goals of promoting healthier eating diets is reducing excess weight among the population.

Other studies that have also taken this approach have found that reduced-energy healthy diets are less expensive than current diets [27, 33, 34] but, in these examples, alcohol beverages and convenience foods represented large proportions of their modelled regular diet cost (53% or more). This is not the case for the current Argentinean diet, where these two categories represented about 19% of the total cost. As has also been found in previous research [31, 35], higher cost-per-energy food categories were more significant contributors to healthier diet budgets compared to current diets, such as fruits and non-starchy vegetables (~ 37% vs. ~ 13%). While meat is the single largest contributor to current diet cost (~ 29%), it also represents a large proportion of the healthy diet budget (~ 25%).

Although the cost of modelled diets is an important variable, it is necessary to consider it in relation to the actual purchasing power of Argentinean families in order to understand its implications for public health. In view of our results and an affordability threshold of 50% of total household income, at least 50% of the Argentinean households could not afford either versions of the healthy diets. Moreover, at least 40% of the population could not afford the current diets. This means that even if they chose products with lower nutritional quality, a large proportion of Argentinean households are not able to afford sufficient food to cover their energy needs. Households classified as poor would have to spend over 70% of their total income in CDs, and all their income to guarantee equal calorie intake through HDs. This situation reveals a high prevalence of food insecurity, which is on itself a strong incentive to minimize energy costs by choosing cheaper, lower quality ingredients and more energy-dense products to reduce the risk of hunger [11].

The differential cost and affordability of healthy vs. unhealthy diets is a key issue to consider when designing public policies to reduce NCDs in Argentina. As has been recommended by PAHO, increasing taxation of less healthy products could be one way to reduce this gap and facilitate consumption of nutrient-rich, less energy-dense foods. Specific taxes on sugar-sweetened beverages in Mexico have already proven to be effective in reducing their consumption [36]. There is also strong evidence supporting governmental subsidies to high quality foods as an effective measure to increase the nutritional quality of regular diets [37]. However, as suggested by our affordability analysis, many households are not able to afford even regular diets. This level of food insecurity is a very serious concern that should be addressed, but it also provides an opportunity for decision-makers to facilitate healthier choices among the population, an opportunity that has not been fully seized. For example, in the context of a still ongoing economic crisis and high inflation rates, the national government implemented ‘Precios Cuidados’, a program developed in collaboration with food manufacturers and retailers to control prices for a basket of mass consumption products. These are a set of reference prices ‘to prevent abuse’ and reduce ‘price dispersion with clear and accurate information’ [38]. However, the products in the program were chosen following criteria that are not coherent with public health objectives. A study conducted in 2019 found that almost 40% of these products had a poor nutritional quality with high content of critical nutrients [39]. This situation also brings to light the lack of coordination between decision makers in different governmental sectors. While there are several governmental programs in place to address food insecurity in Argentina, most of them aim to reduce the risk of immediate hunger and pay little to no attention to broader, public health goals [11].

Although analysing complexities of reducing food insecurity exceed the objectives of this paper, it is clear that an integrated approach is needed, one that brings together different areas of government (finance, public health, education, agricultural policy, etc.). Consumers do not choose products based solely on a utilitarian perspective, to get ‘as many calories as possible for the lowest prices’ [40]. Economic incentives are effective, but insufficient to promote change in food choices if they are not accompanied by other interventions aimed at steering consumers towards better products, such as improving food environments at school, restricting advertising for unhealthy food products, and implementing a front-of-pack label scheme based on nutrient content warnings [41]. Conversely, if diets based on governmental recommendations are unaffordable by over half of the population, these become unattainable ideals, only achievable by the wealthiest Argentineans. In a country that produces enough food to meet the energy intake requirements of 442 million people [11], there is certainly room for improvement.

### Strengths and limitations

This study is one of the first to employ the optimal approach within the INFORMAS framework to assess the cost differential between healthy and current diets in Latin America, and as such can provide useful methodological tools for researchers in the region and crucial information for policy makers at the national and regional levels. By presenting several cost metrics and comparing current, and healthy diets, this study allows a valuable insight into how expensive it is for Argentinean households to increase the quality of their diet.

One of the main limitations of this study relates to data sources. Since the National Nutrition and Health Survey data was significantly outdated (2004–2005) when this study began, we had to turn to the National Household Expenditure Survey (ENGHo) 2012–2013 (2018–2019 was not available yet) to determine the most consumed products and estimate nutrient intake in Argentina. However, this might not be a serious limitation, considering that household expenditure surveys have been used successfully for similar purposes in other studies [42–44]. Product prices were determined using Consumer Price Index data, another governmental data source. These surveys have national coverage and data collection methods are also published, which increases reliability. On the downside, they often include a small number of products or granularity is limited to the food category level. To compensate for these shortcomings, private consultants were engaged, when necessary. Since price data was not available from a single source, inconsistency issues are to be expected due to diverging collection methodologies.

## Conclusions

In average, a reference household must spend almost 32% more money on food to ensure equal energy from a healthy diet than what is currently the norm in Argentinean diets. Moreover, at least 40% of the population could not afford the current diet and this is even worse for the poorer households who would have to spend over 70% of their total income in the CD. Considering that food cost and affordability is one of the main determinants of food choice, this cost gap represents a serious obstacle to the promotion of healthier eating habits in the general population and could also contribute to the high prevalence of excess weight across all age groups, particularly among lower income households. The findings presented here are a key asset for evidence-based policy making, such as the implementation of tailored taxing of food products and governmental food subsidies.

## Data Availability

The datasets used and/or analysed during the current study are available from the corresponding author upon reasonable request.
